# Visual acuity, amblyopia, and vision-related quality of life in preterm adults with and without ROP: results from the Gutenberg prematurity eye study

**DOI:** 10.1038/s41433-022-02207-y

**Published:** 2022-09-15

**Authors:** Achim Fieß, Katrin Greven, Eva Mildenberger, Michael S. Urschitz, Heike M. Elflein, Fred Zepp, Bernhard Stoffelns, Norbert Pfeiffer, Alexander K. Schuster

**Affiliations:** 1 Department of Ophthalmology, University Medical Center of the Johannes Gutenberg University Mainz, Mainz, Germany; 2 Division of Neonatology, Department of Pediatrics, University Medical Center of the Johannes Gutenberg University Mainz, Mainz, Germany; 3 Division of Pediatric Epidemiology, Institute for Medical Biostatistics, Epidemiology and Informatics, University Medical Center of the Johannes Gutenberg University Mainz, Mainz, Germany

**Keywords:** Quality of life, Eye manifestations

## Abstract

**Objectives:**

This study investigated the effects of prematurity and ROP on visual acuity and VRQoL in adults (18–52 years).

**Methods:**

The Gutenberg Prematurity Eye Study is a retrospective cohort study with a prospective ophthalmologic examination. Preterm and full-term participants at an age from 18 to 52 years were included. Distant corrected visual acuity (DCVA) and VRQoL were assessed in participants (892 eyes of 450 individuals aged 28.6 ± 8.6 years, 251 females) grouped into full-term controls (gestational age [GA] at birth ≥37 weeks), preterm participants without ROP and GA 33–36 weeks (group 2), GA 29–32 weeks (group 3), GA ≤ 28 weeks (group 4), non-treated ROP (group 5) and treated ROP (group 6). Main outcome measures were distant corrected visual acuity (DCVA), VRQoL and prevalence of amblyopia.

**Results:**

The DCVA of the better eye correlated (*p* < 0.001) with GA, birth weight, ROP, ROP treatment, and perinatal adverse events and was poorer in both ROP groups. Visual acuity of <20/200 in the better eye was observed in two participants (4.2%) in the ROP group and one person (6.7%) in the treated ROP group. The prevalence of amblyopia increased in the ROP groups. Compared to full-term controls, visual functioning VRQoL scores were lower in preterm individuals independent of ROP while socioemotional VRQoL scores were only lower in the treated ROP group.

**Conclusion:**

Participants with postnatal ROP and its treatment showed decreased visual acuity and VRQol in adulthood, with amblyopia occurring more frequently in more preterm participants with ROP.

## Introduction

Retinopathy of prematurity (ROP) is a vasoproliferative disease occurring in preterm infants and is a major cause of avoidable blindness in childhood [1]. The number of extremely preterm newborns with a high risk for postnatal ROP has increased in recent years [2], hence the pathogenesis of ROP development is well understood. [3] The major risk factors include extremely preterm delivery, high pO_2_ levels during mechanical ventilation, and fluctuations in pO_2_ [4]. It has been demonstrated that prematurity and postnatal ROP occurrence are associated with decreased visual acuity [5–12], higher frequency of refractive error [13–16], strabismus [17–21], and altered ocular geometry in childhood and adolescence [13, 22–26], however, the long-term effects of ROP and extreme prematurity on visual acuity and prevalence of amblyopia in adulthood are less well known. This is of relevance because over 15 million infants are born prematurely annually, thus reduced visual acuity in those subjects is a public health issue and economic burden [27].

The recent population-based Gutenberg Health Study (GHS) investigated the impact of low birth weight as a surrogate marker of prematurity on visual acuity in adults aged 35 to 74 years, reporting that lower birth weight (<2500 g) was associated with decreased visual acuity compared to normal birth weight individuals. However, the gestational age and postnatal ROP occurrence were not reported in this study [28]. Only two larger studies exist assessing visual acuity in former extreme preterm individuals in adulthood. Darlow et al. [29] assessed visual acuity in former preterm newborns (*n* = 229; birth weight ≤1500 g) aged 27 to 29 years who were screened for ROP after birth but were not treated as there was no ROP treatment available at the time, reporting that adults born preterm with postnatal ROP have decreased visual acuity and problems with vision affecting daily life. In a Swedish study, Pétursdóttir et al. [30] examined 59 preterms (≤1500 g) and 44 full-term individuals aged 25 to 29 years, observing that prematurity without ROP also affects visual function. However, 80% of all preterm infants worldwide are moderate or late preterm newborns with a gestational age ≥ 32 weeks, and data about their visual long-term development is lacking. Furthermore, there is no data available about the effects of prematurity stratified for different maturity levels and the postnatal occurrence of ROP and ROP treatment separately. Hence, this study aimed to assess visual function and visual impairment in former moderate (gestational age [GA] 33–36 weeks), very (GA 29–32 weeks), and extreme (GA ≤ 28 weeks) preterm individuals aged 18–52 years with and without ROP compared to full-term controls.

## Materials and methods

### Study population

The Gutenberg Prematurity Eye Study (GPES) is a single-centre cohort study at the University Medical Center of the Johannes Gutenberg University Mainz in Germany (UMCM) that recruits individuals that i) have been born preterm or at term between 1969 and 2002 and ii) were between 18 and 52 years of age at study enrolment. According to these design elements, the study is a retrospective cohort study with a prospective acquisition of follow-up data. Following an invitation algorithm, every former preterm newborn with gestational age at birth (GA) ≤ 32 weeks and every second randomly chosen preterm newborn with a GA 33–36 weeks were approached. For each calendar month (from 1969 to 2002), six randomly selected full-term subjects (three males and three females) with a birth weight between the 10th and 90th percentile were invited to serve as controls as reported earlier [31–37]. The flow chart for eligibility and the effective recruitment efficacy proportion is displayed in Supplementary Fig. 1.

The examinations were conducted between 2019 and 2021, including visual acuity testing and a medical history interview. The medical records of the study participants documenting their perinatal and postnatal history in the UMCM were also reviewed.

Written informed consent was obtained from all participants before their study entry. The GPES complies with Good Clinical Practice (GCP), Good Epidemiological Practice (GEP), and the ethical principles of the Declaration of Helsinki. The study protocol and study documents were approved by the local ethics committee of the Medical Chamber of Rhineland-Palatinate, Germany (reference no. 2019-14161; original vote: 29.05.2019, latest update: 02.04.2020).

### Assessment of pre-, peri- and postnatal medical history

All patient-related information stored at the UMCM was reviewed and the following data were collected: GA (weeks), birth weight (kg), presence of ROP, stage of ROP, ROP treatment, placental insufficiency, preeclampsia, breastfeeding, maternal smoking during pregnancy and perinatal adverse events. For the present study, birth weight percentiles were calculated according to Voigt et al. [38].

### Categorization

For descriptive analysis, participants were grouped into participants born full-term with GA at birth ≥37 completed weeks (group 1), preterm participants with GA 33–36 weeks without ROP (group 2), GA 29–32 weeks without ROP (group 3), GA ≤ 28 weeks without ROP (group 4), GA ≤ 32 and non-treated ROP (group 5) and treated ROP (group 6). In the case that only one eye was affected with ROP, the other non-ROP eye was excluded from the analysis.

### Ophthalmologic examination

A detailed and comprehensive ophthalmologic examination was conducted including testing of visual acuity without correction and distant corrected visual acuity (DCVA) with (ARK-1s, NIDEK, Oculus, Wetzlar, Germany). Intraocular pressure was measured with a non-contact tonometer (NT 2000™, Nidek Co., Japan). Visual acuity was converted from decimal to logMAR according to the medical literature [39]. Red-green colour vision deficiency was assessed by self-report and stereopsis was tested with the Lang II test.

### Vision-related quality of life

Vision-related QoL was assessed with the German version of the National Eye Institute Visual Function Questionnaire (NEI VFQ-25) [40], which was previously validated [40, 41]. The NEI VFQ-25 consists of 25 items (http://www.rand.org/health/surveys_tools/vfq.html, last accessed 2019-07-19). Rasch analysis was performed [42, 43] to transform the raw data into an interval-level scale to generate person-level scores for two traits: the visual functioning scale and the socioemotional scale [44].

### Definition of amblyopia

Participants were judged as having amblyopia according to criteria previously published for a German cohort [45]. Unilateral amblyopia was defined as BCVA in the better eye ≤0.63 with a two-line difference (difference between the visual acuity of the two eyes of at least two lines of vision) or ≤0.5 without such a difference and strabismus or history of strabismus and/or anisometropia ≥1.0 dioptre (spherical, cylindrical, affecting the weaker eye) and/or no other ophthalmological abnormalities that explain limited vision in one participant. Bilateral amblyopia was defined as BCVA ≤ 0.63 in both eyes and binocular hyperopia ≥4.0 dioptre and/or bilateral astigmatism ≥2.0 dioptre and bilateral myopia ≥6.0 dioptre and/or bilateral deprivation and no other ophthalmological abnormalities that explain limited vision [45]. Participants with retinal detachment, cataracts, and other vision-reducing ophthalmic diseases assessed by slit-lamp examination were classified as not amblyopic. Furthermore, all participants were asked about their history of self-reported amblyopia and amblyopia treatment.

### Covariables

The risk factors that may affect the outcome measures, such as gender (female), age (years), GA (weeks), birth weight (kg), birth weight percentile, ROP (yes), ROP treatment (yes), placental insufficiency (yes), preeclampsia (yes), maternal smoking (yes), breastfeeding (yes) and perinatal adverse events (yes) were considered as covariables. Perinatal adverse events were defined according to the German query for quality control of the neonatal clinics: occurrence of intraventricular haemorrhage (at least grade 3 or parenchymal haemorrhage), the occurrence of necrotizing enterocolitis, and moderate or severe bronchopulmonary dysplasia were summarized as adverse events.

### Statistical analysis

The main outcome measure was distant corrected visual acuity (DCVA) in the better eye. Descriptive statistics were computed stratified by the clinical group. Absolute and relative frequencies were calculated for dichotomous parameters, the mean and standard deviation were calculated for approximately normally distributed variables (otherwise median and interquartile range). Spearman’s rank correlation was used to assess the associations between DCVA in the better eye and the above-mentioned covariables. Furthermore, associations between covariables and overall amblyopia were assessed by simple logistic regression analysis. Covariables associated with overall amblyopia in simple regression were further evaluated in a multivariable logistic regression model #1. Here, birth weight, ROP occurrence, and ROP treatment were not considered due to their high correlation with GA. In a multivariable model #2, all relevant covariables of model #1 and postnatal ROP occurrence were investigated to assess the relative independence of these risk factors. This was an explorative study, so no adjustments for multiple testing were performed. Calculations were performed using commercial software (IBM SPSS 20.0; SPSS, Inc., Chicago, IL, USA).

## Results

### Participant characteristics

In the present study, 612 eyes of 310 preterms and 280 eyes of 140 full-term individuals were examined (aged 28.6 ± 8.6 years, 251 females). The different groups are described in Table 1. In the group treated for ROP, seven participants (14 eyes) had treatment with laser coagulation and eight participants (16 eyes) with cryocoagulation. The recruitment efficacy proportion for each group is presented in Supplementary Fig. 1. Furthermore, eight eyes without ROP were excluded in which the fellow eye was affected with postnatal ROP. The participants’ characteristics are described in Table 1. In group 4, one person had retinal breaks in adulthood with a need for retinopexy and two had a history of partial retinal detachment. In the untreated ROP group, one person had bilateral total retinal detachment and another had the same in one eye. In the ROP treated group, two persons had bilateral retinal detachment and one person had a unilateral retinal detachment.

**Table 1:** Characteristics of the sample (*n* = 450) of the GPES stratified by study groups.

	Group 1	Group 2	Group 3	Group 4	Group 5	Group 6
Gestational age	GA ≥ 37	GA 33–36	GA 29–32	GA ≤ 28	GA ≤ 32	GA ≤ 32
		no ROP	no ROP	no ROP	ROP without treatment	ROP with treatment
Participants (*n*)/eyes (*n*)	140/280	137/274	92/184	18/36	48/88	15/30
Gender (Women) (%)	81 (57.9%)	82 (59.9%)	50 (54.3%)	9 (50.0%)	24 (50.0%)	5 (33.3%)
Age (y)	29.9 ± 9.1	29.5 ± 9.1	28.2 ± 8.0	23.4 ± 7.4	25.0 ± 6.0	26.7 ± 2.3
Birth weight (g)	3420 ± 392	2068 ± 464	1559 ± 330	918 ± 197	1057 ± 387	807 ± 244
Birth weight <1500 g (yes)	0 (0%)	13 (9.5%)	38 (41.3%)	18 (100%)	41 (85.4%)	15 (100%)
Birth weight <1000 g (yes)	0 (0%)	0 (0%)	5 (5.4%)	11 (61.1%)	23 (47.9%)	12 (80%)
Birth weight percentile	48.6 ± 21.4	25.2 ± 24.1	45.3 ± 25.0	42.9 ± 25.0	38.2 ± 28.0	24.8 ± 22.5
Gestational age (wks)	39.3 ± 1.3	34.3 ± 0.9	30.6 ± 1.2	26.6 ± 1.5	27.8 ± 2.1	26.7 ± 2.3
(min–max)	(37–43)	(33–36)	(29–32)	(23–28)	(24–32)	(24–32)
ROP stage (1/2/3/4/5)	0/0/0/0/0	0/0/0/0/0	0/0/0/0/0	0/0/0/0/0	32/48/6/0/2	0/6/22/2/0
Perinatal adverse events (yes)^a^	1 (0.7%)	4 (2.9%)	6 (6.5%)	3 (16.7%)	17 (35.4%)	11 (73.3%)
Intraventricular haemorrhage (yes)^b^	0 (0%)	0 (0%)	1 (1.1%)	0 (0%)	2 (4.2%)	1 (6.7%)
Bronchopulmonary dysplasia (yes)^c^	1 (0.7%)	1 (0.7%)	4 (4.3%)	1 (5.6%)	13 (27.1%)	7 (46.7%)
Necrotizing entercolitis (yes)	0 (0%)	3 (2.2%)	1 (1.1%)	2 (11.1%)	4 (8.3%)	6 (40.0%)
Periventricular leukomalacia (yes)	0 (0%)	1 (0.7%)	1 (1.1%)	1 (5.6%)	4 (8.3%)	1 (6.7%)
Preeclampsia (yes)	11 (7.9%)	24 (17.5%)	10 (10.9%)	3 (16.7%)	10 (20.8%)	4 (26.7%)
Placental insufficiency (yes)	2 (1.4%)	16 (11.7%)	2 (2.2%)	1 (5.6%)	3 (6.2%)	0 (0%)
HELLP-syndrome	0 (0%)	6 (4.4%)	1 (1.1%)	0 (0%)	4 (8.3%)	0 (0%)
Maternal smoking (yes)^d^	7 (5.0%)	8 (5.8%)	8 (8.7%)	1 (5.6%)	5 (10.4%)	3 (20%)
Gestational diabetes (yes)	1 (0.7%)	7 (5.1%)	1 (1.1%)	1 (5.6%)	1 (2.1%)	0 (0%)
Breastfeeding (yes)	79 (56.4%)	75 (54.7%)	46 (50.0%)	9 (50.0%)	22 (45.8%)	7 (46.7%)
Ocular parameters						
Spherical equivalent (dioptre) OD	−0.98 ± 2.18	−1.10 ± 2.19	−0.62 ± 2.18	−0.69 ± 2.27	−1.30 ± 2.87	−1.60 ± 2.84
Spherical equivalent (dioptre) OS	−0.97 ± 2.09	−1.18 ± 2.17	−0.81 ± 2.55	−0.41 ± 1.96	−1.74 ± 3.29	−3.38 ± 9.31
Intraocular pressure (mmHg) OD	15.3 ± 2.8	14.6 ± 3.0	15.1 ± 3.2	16.7 ± 3.3	15.3 ± 4.2	18.2 ± 4.3
Intraocular pressure (mmHg) OS	15.2 ± 2.8	14.5 ± 3.1	14.5 ± 2.9	15.2 ± 3.0	15.7 ± 3.7	16.2 ± 3.5

*g* gram, *mm* millimetre, *GA* gestational age, *ROP* retinopathy of prematurity, *y* years, *n* number, *OD* right eye, *OS* left eye.

^a^Perinatal adverse events were defined as occurrence of.

^b^intraventricular haemorrhage (at least grade 3 or parenchymal haemorrhage) and/or occurrence of necrotizing enterocolitis.

^c^and/or bronchopulmonary dysplasia (moderate or severe.)

^d^maternal smoking during pregnancy.

### Descriptive visual acuity measures

**Fig. 1:**
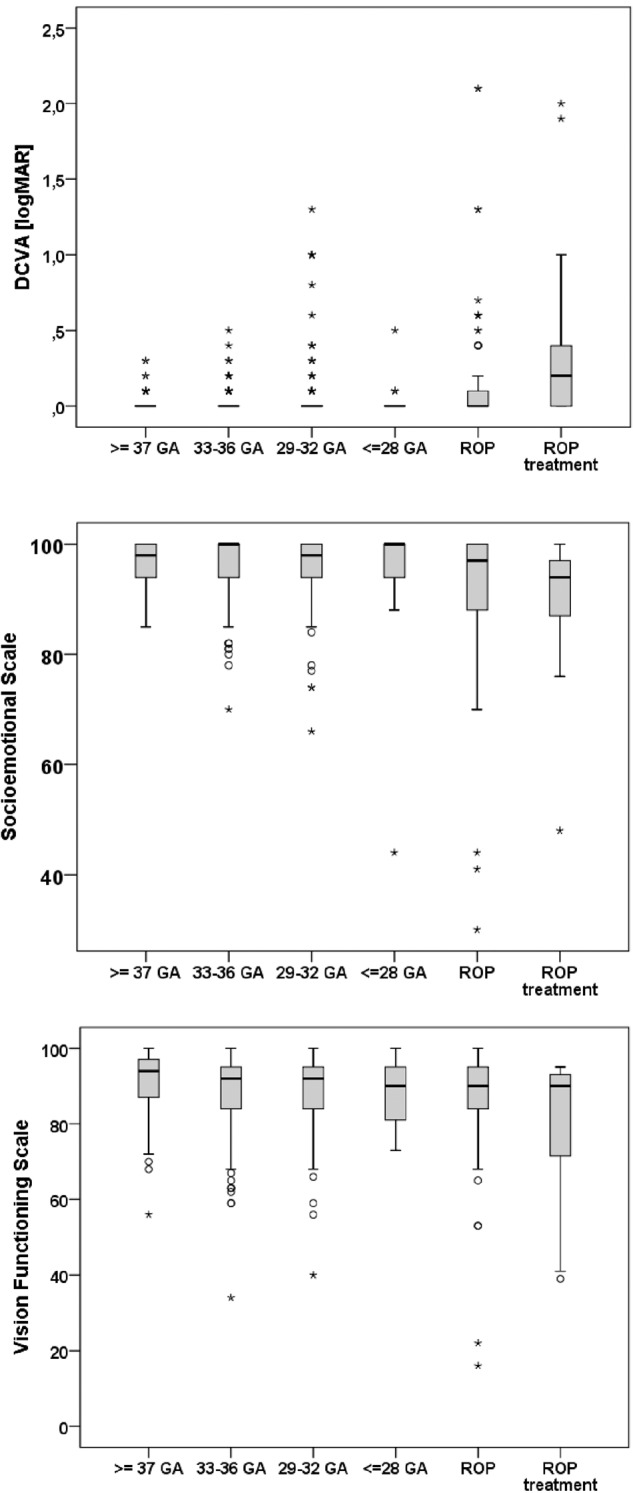
Distant corrected visual acuity, socioemotional scale and vision functioning scale for each group of the GPES sample. GA gestational age, ROP Retinopathy of prematurity.

Table 2 presented uncorrected visual acuity and distant corrected visual acuity. The ROP treated group had the poorest visual acuity (Fig. 1). Visual acuity of <20/200 in the better eye was observed in two participants (4.2%) in the ROP group and one person (6.7%) in the ROP treated group. The more immature the participants at birth, the more likely they reported amblyopia and history of occlusion therapy in childhood, especially for the ROP treated group. The prevalence of unilateral and bilateral amblyopia is presented in Table 2, with a higher prevalence of any amblyopia in the ROP groups. (Table 2).

**Table 2:** Visual acuity, vision functioning and socioemotional scale and amblyopia parameters as well as NEI VFQ-25 items for the GPES sample (*n* = 450) for each study group.

	Group 1	Group 2	Group 3	Group 4	Group 5	Group 6
Gestational age	GA ≥ 37	GA 33–36	GA 29–32	GA ≤ 28	GA ≤ 32	GA ≤ 32
		no ROP	no ROP	no ROP	ROP without treatment	treat. ROP treatment
Participants/eyes (*n*)	140/280	137/274	92/184	18/36	48/88	15/30
Uncorrected visual acuity (logMAR) OD	0.0 (0.0; 0.1)	0.0 (0.0; 0.3)	0.1 (0.0; 0.3)	0.1 (0.0; 0.2)	0.1 (0.0; 0.3)	0.5 (0.0; 0.9)
Uncorrected visual acuity (logMAR) OS	0.0 (0.0; 0.2)	0.0 (0.0; 0.4)	0.1 (0.0; 0.3)	0.0 (0.0; 0.2)	0.1 (0.0; 0.6)	0.3 (0.2; 1.3)
DCVA (logMAR) OD	0.0 (0.0; 0.0)	0.0 (0.0; 0.0)	0.0 (0.0; 0.0)	0.0 (0.0; 0.0)	0.0 (0.0; 0.1)	0.1 (0.0; 0.3)
DCVA (logMAR) OS	0.0 (0.0; 0.0)	0.0 (0.0; 0.0)	0.0 (0.0; 0.0)	0.0 (0.0; 0.0)	0.0 (0.0; 0.1)	0.3 (0.1; 0.8)
Visual acuity better eye (logMAR) OS	0.0 (0.0; 0.0)	0.0 (0.0; 0.0)	0.0 (0.0; 0.0)	0.0 (0.0; 0.0)	0.0 (0.0; 0.0)	0.1 (0.0; 0.3)
Visual acuity worse eye (logMAR) OS	0.0 (0.0; 0.0)	0.0 (0.0; 0.0)	0.0 (0.0; 0.0)	0.0 (0.0; 0.0)	0.0 (0.0; 0.1)	0.4 (0.1; 1.0)
Visual acuity better eye:						
<20/20 (Decimal) (*n* (%))	9 (6.4%)	7 (5.1%)	8 (8.7%)	2 (11.2%)	10 (20.8%)	9 (60%)
<20/40 (Decimal) (*n* (%))	0 (0%)	0 (0%)	4 (4.3%)	0 (0%)	6 (12.5%)	1 (6.7%)
<20/60 (Decimal) (*n* (%))	0 (0%)	0 (0%)	2 (2.2%)	0 (0%)	4 (8.3%)	1 (6.7%)
<20/200 (Decimal) (*n* (%))	0 (0%)	0 (0%)	0 (0%)	0 (0%)	2 (4.2%)	1 (6.7%)
<20/400 (Decimal) (*n* (%))	0 (0%)	0 (0%)	0 (0%)	0 (0%)	1 (2.1%)	1 (6.7%)
Eyes without light perception (*n* (%))	0 (0%)	0 (0%)	1 (1.1%)	0 (0%)	0 (0%)	1 (6.7%)
No stereopsis (Lang test II) (*n* (%))	2 (1.4%)	9 (6.6%)	14 (15.2%)	3 (16.7%)	13 (27.1%)	9 (60.0%)
Red-green color vision deficiency (*n* (%))	0 (0%)	3 (2.2%)	3 (3.3%)	0 (0%)	4 (8.3%)	0 (0%)
Self-reported amblyopia (*n* (%))	10 (7.1%)	8 (5.8%)	15 (16.3%)	3 (16.7%)	13 (27.1%)	9 (60%)
History of amblyopia treatment (*n* (%))	5 (3.6%)	6 (4.4%)	6 (6.5%)	3 (16.7%)	10 (20.8%)	9 (60%)
Unilateral amblyopia at examination (*n* (%))	2 (1.4%)	4 (2.9%)	5 (5.4%)	1 (5.6%)	3 (6.2%)	3 (20%)
Bilateral amblyopia at examination (*n* (%))	1 (0.7%)	2 (1.5%)	5 (5.4%)	0 (0%)	5 (10.4%)	4 (26.7%)
Any amblyopia at examination (*n* (%))	3 (2.1%)	6 (4.4%)	10 (10.9%)	1 (5.6%)	8 (16.7%)	7 (46.7%)
NEI VFQ-25 items						
Car driving (yes) (*n* (%))	127 (90.7%)	123 (89.8%)	86 (93.5%)	12 (66.7%)	32 (66.7%)	8 (53.3%)
Cancelled car driving due to low VA (*n* (%))	0 (0%)	1 (0.7%)	0 (0%)	1 (5.6%)	1 (2.1%)	1 (6.7%)
Difficulties with reading (*n* (%))	25 (17.9%)	35 (25.5%)	22 (23.9%)	4 (22.2%)	15 (31.2%)	7 (46.7%)
Vision related quality of life						
Vision functioning scale	91.6 ± 8.0	88.1 ± 11.2^a^	88.7 ± 10.7^a^	88.4 ± 8.4	85.1 ± 17.9^a^	79.1 ± 18.7^a^
Socioemotional scale	96.9 ± 4.1	96.0 ± 5.9	95.4 ± 7.0	94.1 ± 13.5	90.9 ± 15.9	88.9 ± 13.7^a^

Visual acuity is described as median and interquartile range.

Mann–Whitney-*U*-test was applied to compare VRQoL of the different groups with the full-term control group (reference).

*g* gram, *mm* millimetre, *GA* gestational age, *ROP* retinopathy of prematurity, *y* years, *n* number, *OD* right eye, *OS* left eye, *DCVA* distant corrected visual acuity.

^a^Statistical difference (*p* < 0.05) compared to the control group.

### Vision-related quality of life

Table 2 and Fig. 1 present the VRQoL results, showing that compared to the full-term control group, vision scores in the functioning scale were lower in the participants born moderately preterm (33–36 GA without ROP; group 2, *p* = 0.012), very preterm (29–32 GA without ROP; group 3, *p* = 0.038), participants with postnatal ROP occurrence without treatment (group 5, *p* = 0.012) and participants with postnatal ROP treatment (group 6, *p* = 0.002). Significantly lower scores concerning the socioemotional scale were only observed in participants with postnatal ROP treatment (group 6) compared to the control subjects (*p* = 0.002).

### Association analyses

DCVA was associated with GA (*r* = −0.18; *p* < 0.001), lower birth weight (*r* = −0.18; *p* < 0.001), ROP (*r* = 0.29; *p* < 0.001); ROP treatment (*r* = 0.32; *p* < 0.001), and perinatal adverse events (*r* = 0.29; *p* < 0.001) (Table 3). Overall amblyopia was associated with low gestational age, low birth weight, postnatal ROP occurrence and treatment, placental insufficiency, and perinatal adverse events. In multivariable analysis, amblyopia was related to low GA [B = 0.88 (95% CI: 0.79; 0.99), *p* = 0.029] and perinatal adverse events [*B* = 3.68 (95% CI: 1.38; 9.84), *p* = 0.009] but not to ROP occurrence (Table 4).

**Table 3:** Association analyses of the distant corrected visual acuity of the better eye (*n* = 450) for the GPES sample.

DCVA better eye [logMAR]	Univariate	
	B [95% CI]	*p*
	Spearman correlation	
Gestational age (weeks)	−0.18	<0.001
Birth weight (kg)	−0.18	<0.001
Birth weight percentile	−0.05	0.34
ROP (yes)	0.29	<0.001
ROP treatment (yes)	0.32	<0.001
Perinatal adverse events (yes)	0.29	<0.001
Smoking during pregnancy (yes)	0.03	0.54
Preeclampsia (yes)	0.00	0.93
Breastfeeding (yes)	−0.03	0.48
Placental insufficiency (yes)	0.00	0.98

Spearman correlation test.

*B* Beta, *CI* Confidence interval.

**Table 4:** Association analyses of the prevalence of amblyopia (*n* = 450) in the GPES sample.

	univariable		model 1		model 2	
	OR [95% CI]	*p*	OR [95% CI]	*p*	OR [95% CI]	*p*
Any amblyopia [yes]						
Gestational age (weeks)	0.81 (0.74; 0.89)	<0.001	0.86 (0.78; 0.96)	0.005	0.88 (0.79; 0.99)	0.029
Birth weight (kg)	0.37 (0.23; 0.61)	<0.001	–	–	–	–
Birth weight percentile	1.00 (0.98; 1.01)	0.50	–	–	–	–
ROP (yes)	5.47 (2.56; 11.67)	<0.001	–	–	1.36 (0.48; 3.88)	0.57
ROP treatment (yes)	11.14 (3.69; 33.58)	<0.001	–	–	–	–
Perinatal adverse events (yes)	8.52 (3.83; 18.96)	<0.001	3.81 (1.50; 9.72)	0.005	3.68 (1.38; 9.84)	0.009
Smoking during pregnancy (yes)	0.80 (0.18; 3.54)	0.77	–	–	–	–
Preeclampsia (yes)	1.34 (0.53; 3.41)	0.54	–	–	–	–
Breastfeeding (yes)	0.54 (0.27; 1.11)	0.094	–	–	–	–
Placental insufficiency (yes)	3.27 (1.01; 10.59)	0.048	2.75 (0.74; 10.23)	0.13	–	–

Univariate model: adjusted for age and sex.

Model 1: Multivariable model with inclusion of univariable associated parameters with adjustment for age and sex.

Model 2: Multivariable model with inclusion of associated parameters of model 1 and additional inclusion of ROP occurrence. Because of a high collinearity with gestational age the parameter birth weight was not included in the multivariable models.

*OR* Odds ratio, *CI* Confidence interval.

## Discussion

In the present study, severe vision loss and amblyopia occurred in individuals born preterm with postnatal treated or non-treated ROP. Nearly all preterm groups had a lower vision-related quality of life in the visual functioning scale compared to the full-term control group. Furthermore, the more preterm the participants at birth, the more likely they were to have impaired stereopsis.

Various studies have assessed the effects of extreme prematurity and ROP occurrence and treatment on visual function in childhood, [5–12] reporting increased ophthalmologic disorders and reduced visual function in schoolchildren born preterm compared to full-term controls. In two of these population-based cohorts, follow-up examinations were performed in adulthood. Darlow et al. [29] examined participants aged 27 to 29 years who had prospectively been screened for ROP during the neonatal period, observing that preterm birth and ROP had an impact on long-term visual morbidity. Furthermore, they reported that untreated ROP of stage 2 or more not leading to retinal detachment had a significant effect on reduced visual acuity. None of their study participants received postnatal ROP treatment because they were born before treatment was introduced in New Zealand. The participants born with a very low birth weight experienced more frequent difficulties with daily life activities and were less likely to drive a car. This is in line with our study, whereby participants born extremely preterm (GA ≤ 28 weeks) without ROP and participants with ROP were less likely to drive a car because of problems with visual acuity.

Saigal et al. [46] prospectively followed up 166 Canadian preterm infants (birth weight <1000 g) born between 1977 and 1982, observing that six individuals born preterm (4%) experienced retinal detachment until the age of 23 years. Darlow et al. [29] also observed retinal detachment in two participants at 16 years of age. In the present study, we observed that seven of 310 (2.3%) preterm participants had a history of retinal detachment in at least one eye. However, it must be considered that some of these participants were born before available ROP treatment.

In a Swedish study, Pétursdóttir et al. [30] examined 44 former full-term and 59 former preterm individuals now aged between 25 and 29 years, reporting lower distant and near visual acuity, impaired visual field examinations (mean deviation), and lower contrast sensitivity in the better eye in the preterm compared to the full-term control group. Interestingly, distant visual acuity and contrast sensitivity remained reduced in preterm participants after the exclusion of those with previous ROP and neurological complications. The prevalence of visual acuity worse than 20/20 in 10% of preterm and 2.3% of full-term participants was lower compared to the results of Darlow et al. [29] reporting a prevalence of 33% in preterm and 13% in full-term participants. However, both studies did not stratify their data for the maturity of preterm participants without ROP. In our study, we demonstrated that the prevalence of DCVA poorer than 20/20 in the better eye was comparable between full-term controls and moderate, very, and extreme preterm participants without postnatal ROP. However, it is noteworthy that in the present study nearly all preterm groups had decreased VRQoL regarding the visual functioning scale while scoring in the socioemotional scale was only reduced in the ROP group requiring treatment compared to the full-term control group. This indicates that severe ROP requiring treatment is a very important parameter for the long-term development of visual acuity and vision function affecting VRQoL. This is supported by the finding that the prevalence of DCVA poorer than 20/20 was 21% in the group with postnatal ROP without treatment and 60% in the treated ROP group.

While the effects of preterm birth on visual function in childhood are known, there is a lack of data regarding the prevalence of amblyopia. Furthermore, the definitions for amblyopia and the inclusion criteria in studies differ in the medical literature which leads to difficulties in comparing previous reports. Schalij-Delfos et al. [47] reported a 10% prevalence of amblyopia in participants with a GA between 33–36 weeks, 22% in participants with a GA between 28–32 weeks, and 32% in participants with GA < 28 weeks. However, little is known about the prevalence of amblyopia in adulthood. In the large population-based GHS, the authors reported a prevalence of 5% amblyopia for the general German population aged 35–44 years, [45] which is comparable to the prevalence of amblyopia in the full-term and moderate preterm groups in our study. Moreover, the present study extends the medical literature demonstrating that both ROP groups showed a significantly increased prevalence of amblyopia, highlighting the long-term effects of prematurity and ROP occurrence and treatment in these individuals. Furthermore, the association of perinatal adverse events with amblyopia in the multivariable model indicates that adverse events during perinatal development might be another important predictor for amblyopia in addition to the immaturity of the newborn.

Different mechanisms may explain the effects of prematurity and associated factors on visual acuity and VRQoL in adulthood. Our data enabled a comprehensive analysis of the different parameters potentially associated with reduced DCVA in the better eye. We observed a univariate correlation between DCVA and low gestational age, birth weight, ROP occurrence, ROP treatment, and perinatal adverse events. In contrast to Pétursdóttir et al. [30] who hypothesized that particularly prematurity and not ROP occurrence or treatment are important for visual dysfunction, we found reduced visual function mainly in ROP participants and particularly in ROP treated participants. One reason for reduced visual function in preterm persons and particularly in individuals with and without ROP treatment may be the postnatal occurrence of retinal detachment and structural changes in macula morphology. In a large cross-sectional study of more than 500 preterm and full-term participants, the authors reported that the more preterm the participants were born, the more altered the foveal structure. ROP was found to be an additional independent factor leading to increased foveal thickness [23] potentially affecting visual function. Furthermore, in an MRI study of an extremely low birth weight (<1000 g) cohort, altered optical radiation and visual cortex were observed in comparison to a control group which may also be one reason for visual dysfunction in former preterm newborns [48].

### Strengths and limitations

The present study had some limitations. It was a single-centre hospital-based study and some people declined to take part in the study examination. Furthermore, there was a high number of participants that were not contactable and it is possible that subjects with greater morbidity may have been more difficult to recruit. As a consequence, there is the possibility that the study is biased towards those with less severe morbidity, and may rather underrepresent the impact of prematurity and ROP on the outcome measures. It is important to note that only a few participants were included with advanced ROP stages and the need for ROP treatment. Furthermore, it is noteworthy that a high proportion of participants treated for ROP had cryotherapy, which is now rarely performed in developed countries and current practice may deliver different long-term outcomes. This issue also limits the strengths of conclusions, as it leaves only seven subjects who were treated for ROP in a way consistent with current practice. This should be considered when the effects of advanced stages of ROP and ROP treatment are interpreted in the present study. As extreme prematurity and low birth weight are established risk factors for cerebral visual impairment, this may have a key role in contributing to visual impairment in the present cohort. Although we assessed intraventricular haemorrhage and periventricular leucomalacia we can not fully exclude the presence of other cerebral lesions and cognitive delay, thus we can not adjust for and this could contribute to visual impairment particularly in the ROP groups. Furthermore, most participants were Caucasians, so the findings cannot be generalized to other ethnic groups. Another limitation is that participants with low visual acuity are less likely to participate in our study which may reduce the representativeness.

The main strength of the present study is the examination of a large sample size including various preterm participants. Thus, this study enables a unique and first view of the effects of different degrees of prematurity independent of ROP. The strengths of this study are the detailed and comprehensive assessment of perinatal data from medical charts. Furthermore, visual acuity measurements were conducted by investigators blind to the participants’ birth history, thus, investigator-dependent bias was unlikely. Each examination of visual function was also performed according to strict standardized operating procedures to avoid examiner-dependent variations.

## Conclusions

In conclusion, the postnatal occurrence of advanced stages of ROP requiring treatment leads to decreased visual acuity and vision-related quality of life in adults, while adults born very and extremely preterm without ROP have fewer long-term effects on visual function. The more immature the participants at birth, the more likely the occurrence of amblyopia, particularly in subjects with advanced stages of ROP requiring treatment had occurred. These results highlight that in the perinatal setting of subjects born preterm, all efforts should be undertaken to avoid ROP occurrence and treatment. All preterm children should be regularly screened for amblyopia to adequately treat and prevent life-long consequences, as amblyopia and impaired stereopsis were frequently detected in adults born preterm (≤32 weeks).

## Summary

### What was known before

- Preterm delivery and postnatal ROP occurrence are associated with reduced visual acuity in infancy and childhood, however, the perinatal long-term effects on visual function, amblyopia, and vision-related quality of life in adulthood are less well known.
- We investigated to what extent different degrees of prematurity, ROP and associated factors may lead to reduced visual function in adults aged 18–52 years.

### What this study adds

- Distant corrected visual acuity of the better eye was correlated with gestational age, birth weight, ROP, ROP treatment, and perinatal adverse events (all *p* < 0.001).
- Amblyopia was most frequently found in the ROP groups. Compared to full-term controls, visual functioning scores were lower in all preterm individuals independent of ROP while socioemotional scores were lower only in the ROP treated group.

## Supplementary information

Supplementary Figure 1: Study design of the Gutenberg Prematurity Eye Study (GPES) Legend: PT-preterm; w - weeks

## Data Availability

Access to data, responsibility, and analysis. AF had full access to all the data in the study and takes responsibility for the integrity of the data and the accuracy of the data analysis. Statistical analyses were performed by AF. The analysis presents clinical data of a cohort. This project constitutes a major scientific effort with high methodological standards and detailed guidelines for analysis and publication to ensure scientific analyses are on the highest level. Therefore, data are not made available for the scientific community outside the established and controlled workflows and algorithms. To meet the general idea of verification and reproducibility of scientific findings, we offer access to data at the local database upon request at any time. Interested researchers make their requests to the coordinating PI of the GPES (AF; achim.fiess@unimedizin-mainz.de). More detailed contact information is available at the homepages of the UM (www.unimedizin-mainz.de).

## References

[1] Kong L, Fry M, Al-Samarraie M, Gilbert C, Steinkuller PG (2012). An update on progress and the changing epidemiology of causes of childhood blindness worldwide. J AAPOS.

[2] Blencowe H, Lawn JE, Vazquez T, Fielder A, Gilbert C (2013). Preterm-associated visual impairment and estimates of retinopathy of prematurity at regional and global levels for 2010. Pediatr Res.

[3] Hellström A, Smith LE, Dammann O (2013). Retinopathy of prematurity. Lancet (Lond, Engl).

[4] Kim SJ, Port AD, Swan R, Campbell JP, Chan RVP, Chiang MF (2018). Retinopathy of prematurity: a review of risk factors and their clinical significance. Surv Ophthalmol.

[5] Dobson V, Quinn GE, Summers CG, Hardy RJ, Tung B (2006). Visual acuity at 10 years in Cryotherapy for Retinopathy of Prematurity (CRYO-ROP) study eyes: effect of retinal residua of retinopathy of prematurity. Arch Ophthalmol (Chic, Ill: 1960).

[6] Holmstrom G, Larsson E (2008). Long-term follow-up of visual functions in prematurely born children-a prospective population-based study up to 10 years of age. J AAPOS.

[7] Holmstrom GE, Kallen K, Hellstrom A, Jakobsson PG, Serenius F, Stjernqvist K (2014). Ophthalmologic outcome at 30 months’ corrected age of a prospective Swedish cohort of children born before 27 weeks of gestation: the extremely preterm infants in sweden study. JAMA Ophthalmol.

[8] O’Connor AR, Stephenson TJ, Johnson A, Tobin MJ, Ratib S, Moseley M (2004). Visual function in low birthweight children. Br J Ophthalmol.

[9] Fieß A, Kolb-Keerl R, Elflein HM, Schuster AK, Knuf M, Kirchhof B (2019). Evaluation of ophthalmic follow-up care of former pre-term and full-term infants aged from 4 to 10 years in Germany - Results of the Wiesbaden Prematurity Study (WPS). Klinische Monatsblatter fur Augenheilkd.

[10] Darlow BA, Clemett RS, Horwood LJ, Mogridge N (1997). Prospective study of New Zealand infants with birth weight less than 1500 g and screened for retinopathy of prematurity: visual outcome at age 7-8 years. Br J Ophthalmol.

[11] Fledelius HC (1996). Pre-term delivery and subsequent ocular development. A 7-10 year follow-up of children screened 1982-84 for ROP. 1) Visual function, slit-lamp findings, and fundus appearance. Acta ophthalmologica Scandinavica.

[12] Fledelius HC, Bangsgaard R, Slidsborg C, laCour M (2015). Refraction and visual acuity in a national Danish cohort of 4-year-old children of extremely preterm delivery. Acta ophthalmologica.

[13] Fieß A, Kolb-Keerl R, Knuf M, Kirchhof B, Blecha C, Oberacher-Velten I (2017). Axial length and anterior segment alterations in former preterm infants and full-term neonates analyzed with Scheimpflug imaging. Cornea.

[14] Cook A, White S, Batterbury M, Clark D (2008). Ocular growth and refractive error development in premature infants with or without retinopathy of prematurity. Investigative Ophthalmol Vis Sci.

[15] Larsson EK, Rydberg AC, Holmstrom GE (2003). A population-based study of the refractive outcome in 10-year-old preterm and full-term children. Arch Ophthalmol (Chic, Ill: 1960).

[16] O’Connor AR, Stephenson TJ, Johnson A, Tobin MJ, Ratib S, Fielder AR (2006). Change of refractive state and eye size in children of birth weight less than 1701 g. Br J Ophthalmol.

[17] Fieß A, Kolb-Keerl R, Schuster AK, Knuf M, Kirchhof B, Muether PS (2017). Prevalence and associated factors of strabismus in former preterm and full-term infants between 4 and 10 Years of age. BMC Ophthalmol.

[18] Bremer DL, Palmer EA, Fellows RR, Baker JD, Hardy RJ, Tung B (1998). Strabismus in premature infants in the first year of life. Cryotherapy for Retinopathy of Prematurity Cooperative Group. Arch Ophthalmol (Chic, Ill: 1960).

[19] Gursoy H, Basmak H, Bilgin B, Erol N, Colak E (2014). The effects of mild-to-severe retinopathy of prematurity on the development of refractive errors and strabismus. Strabismus.

[20] Holmstrom G, el Azazi M, Kugelberg U (1999). Ophthalmological follow up of preterm infants: a population based, prospective study of visual acuity and strabismus. Br J Ophthalmol.

[21] O’Connor AR, Stephenson TJ, Johnson A, Tobin MJ, Ratib S, Fielder AR (2002). Strabismus in children of birth weight less than 1701 g. Arch Ophthalmol (Chic, Ill: 1960).

[22] Fieß A, Christian L, Janz J, Kolb-Keerl R, Knuf M, Kirchhof B (2017). Functional analysis and associated factors of the peripapillary retinal nerve fibre layer in former preterm and full-term infants. Br J Ophthalmol.

[23] Fieß A, Janz J, Schuster AK, Kolb-Keerl R, Knuf M, Kirchhof B (2017). Macular morphology in former preterm and full-term infants aged 4 to 10 years. Graefe’s Arch Clin Exp Ophthalmol = Albrecht von Graefes Arch fur klinische und experimentelle Ophthalmologie.

[24] Fieß A, Schuster AK, Kolb-Keerl R, Knuf M, Kirchhof B, Muether PS (2017). Corneal aberrations in former preterm infants: results from the Wiesbaden prematurity study. Investigative Ophthalmol Vis Sci.

[25] Fieß A, Christian L, Kolb-Keerl R, Knuf M, Kirchhof B, Muether PS (2016). Peripapillary choroidal thickness in former preterm and full-term infants aged from 4 to 10 years. Investigative Ophthalmol Vis Sci.

[26] Fieß A, Schuster AK, Pfeiffer N, Nickels S (2017). Association of birth weight with corneal power in early adolescence: results from the National Health and Nutrition Examination Survey (NHANES) 1999-2008. PloS one.

[27] Chawanpaiboon S, Vogel JP, Moller AB, Lumbiganon P, Petzold M, Hogan D (2019). Global, regional, and national estimates of levels of preterm birth in 2014: a systematic review and modelling analysis. Lancet Glob health.

[28] Fieß A, Schuster AK, Nickels S, Elflein HM, Schulz A, Beutel ME (2019). Association of low birth weight with myopic refractive error and lower visual acuity in adulthood: results from the population-based Gutenberg Health Study (GHS). Br J Ophthalmol.

[29] Darlow BA, Elder MJ, Kimber B, Martin J, Horwood LJ (2018). Vision in former very low birthweight young adults with and without retinopathy of prematurity compared with term born controls: the NZ 1986 VLBW follow-up study. Br J Ophthalmol.

[30] Pétursdóttir D, Holmström G, Larsson E (2020). Visual function is reduced in young adults formerly born prematurely: a population-based study. Br J Ophthalmol.

[31] Fieß A, Fauer A, Mildenberger E, Urschitz MS, Elflein HM, Zepp F, et al. Refractive error, accommodation and lens opacification in adults born preterm and full-term: Results from the Gutenberg Prematurity Eye Study (GPES). Acta ophthalmologica. 2022. Online ahead of print. 10.1111/aos.15116 35297183

[32] Fieß A, Gißler S, Fauer A, Riedl JC, Mildenberger E, Urschitz MS, et al. Short report on retinal vessel metrics and arterial blood pressure in adult individuals born preterm with and without retinopathy of prematurity: results from the Gutenberg Prematurity Eye Study. Acta ophthalmologica. 2022. Online ahead of print. 10.1111/aos.15132 35338589

[33] Fieß A, Gißler S, Mildenberger E, Urschitz MS, Fauer A, Elflein HM, et al. Anterior chamber angle in adults born extremely, very, and moderately preterm with and without retinopathy of prematurity-results of the gutenberg prematurity eye study. Children (Basel, Switzerland). 2022;9:281. 10.3390/children9020281 PMC8869987 35205001

[34] Fieß A, Gißler S, Mildenberger E, Urschitz MS, Zepp F, Hoffmann EM, et al. Optic nerve head morphology in adults born extreme, very and moderate preterm with and without retinopathy of prematurity: Results from the Gutenberg Prematurity Eye Study. Am J of Ophthalmol. 2022;239:212–22. 10.1016/j.ajo.2022.03.005 35288076

[35] Fieß A, Hufschmidt-Merizian C, Gißler S, Hampel U, Mildenberger E, Urschitz MS (2022). Dry eye parameters and lid geometry in adults born extremely, very, and moderately preterm with and without ROP: results from the Gutenberg prematurity eye study. J Clin Med.

[36] Fieß A, Nauen H, Mildenberger E, Zepp F, Urschitz MS, Pfeiffer N, et al. Ocular geometry in adults born extremely, very and moderately preterm with and without retinopathy of prematurity: results from the Gutenberg Prematurity Eye Study. Br J of Ophthalmol. 2022:bjophthalmol-2021-320907. Online ahead of print. 10.1136/bjophthalmol-2021-320907 PMC10359553 35273019

[37] Fieß A, Pfisterer A, Gißler S, Korb C, Mildenberger E, Urschitz MS et al. Retinal thickness and foveal hypoplasia in adults born preterm with and without retinopathy of prematurity–The Gutenberg Prematurity Eye Study. Retina. 2022: 10.1097/IAE.0000000000003501. 10.1097/IAE.0000000000003501 35994585

[38] Voigt M, Fusch C, Olbertz D (2006). Analyse des Neugeborenenkollektivs der Bundesrepublik Deutschland 12. Mitteilung: Vorstellung engmaschiger Perzentilwerte (-kurven) für die Körpermaße Neugeborener. Geburtsh Frauenheilk.

[39] Bach M, Kommerell G (1998). [Determining visual acuity using European normal values: scientific principles and possibilities for automatic measurement]. Klinische Monatsblatter fur Augenheilkd.

[40] Mangione CM, Lee PP, Gutierrez PR, Spritzer K, Berry S, Hays RD (2001). Development of the 25-item National Eye Institute Visual Function Questionnaire. Arch Ophthalmol (Chic, Ill: 1960).

[41] Finger RP, Wiedemann P, Blumhagen F, Pohl K, Holz FG (2013). Treatment patterns, visual acuity and quality-of-life outcomes of the WAVE study - a noninterventional study of ranibizumab treatment for neovascular age-related macular degeneration in Germany. Acta ophthalmologica.

[42] Pesudovs K, Gothwal VK, Wright T, Lamoureux EL (2010). Remediating serious flaws in the National Eye Institute Visual Function Questionnaire. J cataract refractive Surg.

[43] Dougherty BE, Bullimore MA (2010). Comparison of scoring approaches for the NEI VFQ-25 in low vision. Optom Vis Sci: Off Publ Am Acad Optom.

[44] Nickels S, Schuster AK, Singer S, Wild PS, Laubert-Reh D, Schulz A (2017). The National Eye Institute 25-Item Visual Function Questionnaire (NEI VFQ-25) - reference data from the German population-based Gutenberg Health Study (GHS). Health Qual life outcomes.

[45] Elflein HM, Fresenius S, Lamparter J, Pitz S, Pfeiffer N, Binder H (2015). The prevalence of amblyopia in Germany: data from the prospective, population-based Gutenberg Health Study. Dtsch Arzteblatt Int.

[46] Saigal S, Stoskopf B, Boyle M, Paneth N, Pinelli J, Streiner D (2007). Comparison of current health, functional limitations, and health care use of young adults who were born with extremely low birth weight and normal birth weight. Pediatrics.

[47] Schalij-Delfos NE, de Graaf ME, Treffers WF, Engel J, Cats BP (2000). Long term follow up of premature infants: detection of strabismus, amblyopia, and refractive errors. Br J Ophthalmol.

[48] Kelly CE, Cheong JL, Molloy C, Anderson PJ, Lee KJ, Burnett AC (2014). Neural correlates of impaired vision in adolescents born extremely preterm and/or extremely low birthweight. PloS one.

