# Supplementary figures and images for: Visual acuity, amblyopia, and vision-related quality of life in preterm adults with and without ROP: results from the Gutenberg prematurity eye study
Source: Eye (Lond). 2022 Sep 15;37(9):1794–801. doi: 10.1038/s41433-022-02207-y (PMC10275879; doi:10.1038/s41433-022-02207-y)

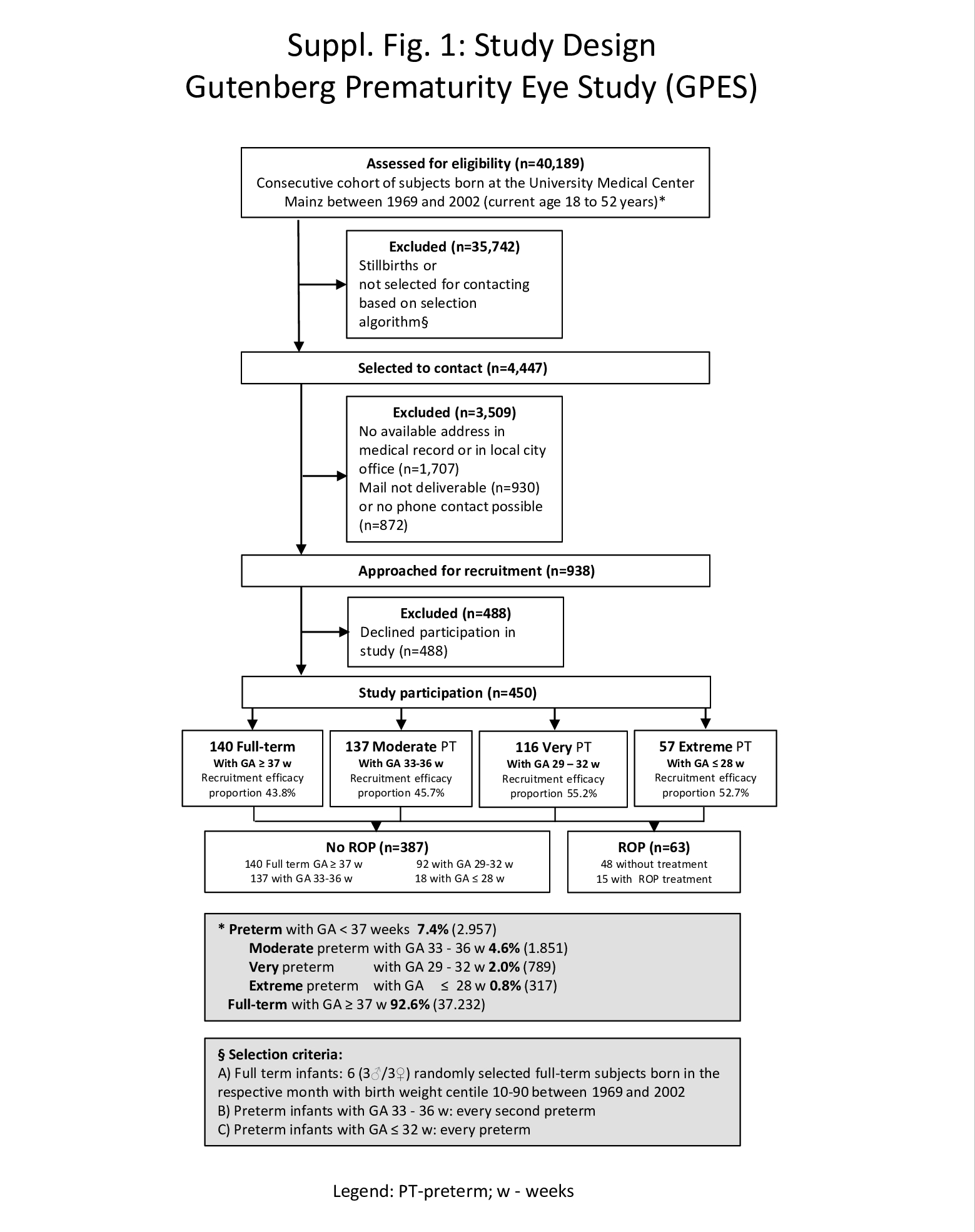

Supplement: Supplementary file 1 — Supplementary Figure 1: Study design of the Gutenberg Prematurity Eye Study (GPES) Legend: PT-preterm; w - weeks [file 41433_2022_2207_MOESM1_ESM.tif]
